# Prevalence of Bronchial Asthma in Indian Children

**DOI:** 10.4103/0970-0218.58389

**Published:** 2009-10

**Authors:** Ranabir Pal, Sanjay Dahal, Shrayan Pal

**Affiliations:** Department of Community Medicine, Sikkim-Manipal Institute of Medical Sciences (SMIMS) and Central Referral Hospital (CRH), 5^th^ Mile, Tadong, Gangtok, Sikkim - 737 102, India; 1 Department of Chemistry, School of Basic and Applied Sciences, Sikkim-Manipal Institute of Technology Campus, Majitar, Sikkim-737 132, India; 2 MBBS Student, Sikkim-Manipal Institute of Medical Sciences (SMIMS) and Central Referral Hospital (CRH), Sikkim - 737 102, India

**Keywords:** Bronchial asthma, children, prevalence

## Abstract

**Background:**

The prevalence of childhood bronchial asthma and allergic disease has increased in developed countries. Studies have identified asthma among Indian children. Still, there is paucity of information on the overall prevalence of childhood asthma in India.

**Objective:**

To assess time trends and the overall prevalence rate of bronchial asthma among Indian children.

**Materials and Methods:**

Literature search for data sources was done through an extensive search in indexed literatures and website-based population survey reports. Fifteen epidemiological studies were identified on the development of asthma in Indian children from 300 potentially relevant articles. A broad criterion to define both allergic and non-allergic descriptions of asthma in Indian children was formed. Moreover, in the absence of universally accepted criteria by reporting of prevalence by researchers, weighted average data was considered during calculations of prevalence rates, irrespective of the criteria for diagnosis. Statistical analyses used were mean and median.

**Results:**

Wide differences in samples, primary outcome variables, lack of consistency in age category, rural–urban variation, criteria for positive diagnosis, and study instruments confounded the outcome variables. The mean prevalence was 7.24 ± SD 5.42. The median prevalence was 4.75% [with IQR = 2.65 − 12.35%]. Overall weighted mean prevalence was found to be 2.74. Childhood asthma among children 13 – 14 years of age was lower than the younger children (6 – 7 years of age). Urban and male predominance with wide inter-regional variation in prevalence was observed.

**Conclusions:**

Our findings indicate that the burden of bronchial asthma in Indian children is higher than was previously understood.

## Introduction

The prevalence of Bronchial Asthma has increased continuously since the 1970s, and now affects an estimated 4 to 7% of the people worldwide. Childhood Bronchial Asthma varies widely from country to country. At the age of six to seven years, the prevalence ranges from 4 to 32%. The same range holds good for ages 13 and 14. UK has the highest prevalence of severe Bronchial Asthma in the world.(1) It has also increased the number of preventable hospital emergency visits and admissions. Apart from being the leading cause of hospitalization for children, it is one of the most important chronic conditions causing elementary school absenteeism.(2,3) Childhood Bronchial Asthma has multifactor causation. Geographical location, environmental, racial, as well as factors related to behaviors and life-styles are associated with the disease.(4–6)

Several studies had identified the prevalence of childhood asthma among Indian children.(7–20) However, the true nature of these findings remains confounded in many studies. To look for sources of potential bias and to try and uncover the consistent patterns of prevalence, a systematic review on bronchial asthma among Indian children was conducted by an extensive array of data.

## Materials and Methods

We attempted an extensive collection of survey results by different sources in which bronchial asthma among Indian children was reported, including meeting presentations and personal communications. Through an extensive website-scanned search in indexed literatures and study reports, we identified 15 epidemiological studies of the development of asthma in Indian children from 300 potentially relevant articles. All published articles in indexed journals available from various institutional libraries of India, and websites on epidemiological studies on bronchial asthma among Indian children published since 1966, were included in this study. The studies were identified by searching Pubmed-entrez and abstracts from scientific meetings (1985 – 2007) also. Reviews of citations and reference lists were performed to identify additional eligible studies. The search terms included bronchial asthma, Indian children, prevalence, asthmatic bronchitis, wheeze, wheezy bronchitis, and reactive airway disease. Where possible, sources were contacted for further information on survey data not readily available in the public domain. Manual searches were conducted from review articles and previous meta-analyses. We also contacted authors for additional information or for translations from languages other than English.

## Selection criteria

We developed few criteria to select studies from among peer-reviewed articles. First, we used broad criteria to define both allergic and non-allergic asthma in Indian children. Second, we sought to include all studies that identified cases of asthma in their analyses by criteria other than symptoms of wheeze alone. Third, our definition included asthma, which was ever or currently recognized by doctor diagnosis or by a set of symptoms that were recognized criteria for diagnosing asthma, in addition to wheezing. Fourth, this study included asthma identified through response to pilot-tested or standardized questionnaires on respiratory health. Finally, in the absence of universally accepted criteria of reporting of prevalence by researchers, weighted average data had been considered as positive cases during calculations of prevalence rates, irrespective of the criteria of diagnosis.

## Main outcome variable

Prevalence of bronchial asthma among Indian children

### Statistical analysis

We managed data using Microsoft Office Excel 2000 and analyzed the data using SPSS (version 10.0) for Windows to calculate the central tendencies and dispersions.

## Results

The main demographic groups of interest for this analysis were Indian children. On data abstraction and synthesis, we observed wide differences in samples and primary outcome variables in the studies. To be more precise heterogeneity varied in age groups, criteria for positive diagnosis, and study instruments. Thereby, results varied to a large extent with regard to the overall prevalence in a majority of studies, with few studies using different parameters, namely, past, ever, current, exercise-induced, and cold. Lack of consistency in case definition within studies and age category altered the summary prevalence in our study. In the absence of universally accepted criteria of reporting of prevalence by researchers, the data was considered as positive cases during calculations of prevalence rates, irrespective of criteria of diagnosis.

The data series [Table 1] had a range of 0.89 to 15.70 with Kurtosis of - 1.90 and skewness of 0.39. The mean prevalence was 7.24 with standard deviation of 5.42. This indicates that the outcome data of prevalence studies reported over this period is widely dispersed.

**Table 1:** Prevalence of bronchial asthma in Indian children

Year	Investigators	Study instruments/Study subjects	Total number of study subjects and age group	Prevalence of childhood bronchial asthma (%)
1966	Vishwanathan R, Prasad M, Thakur AK *et al.*	Patna urban population: A random morbidity survey.	Children < 9 years	0.2% in children below nine years
1998	Multicentric (*ISAAC Steering Committee,*	ISAAC Questionnaire, school-based	37,171 6–7 and 13-14 years	6.0% (Wheeze) 4.5% (Ever asthma) (frequency 1673)
1998	Chhabra SK, Gupta CK, Chhabra P, Rajpal S	Questionnaire-based study	2609 (4 – 17) years	15.7 (frequency 410)
1999	Chhabra K, Gupta CK, Chhabra P, Rajpal S	Questionnaire-based study, Delhi, school-based	19,456 (5 – 17) years	3.4% (Past wheeze) 2.4% (Cold Associated) 12.8% (M) 10.7% (F) 2.1% (Exercise Induced) 11.9% (Current wheeze) (frequency 2315)
2001	Gupta D, Aggarwal AN, Kumar R, Jindal SK	IUATLD based previously standardized questionnaire against physician diagnosis, Chandigarh, school based	4367 boys (9 – 20) years	2.6 (frequency 114)
2001	Gupta D, Aggarwal AN, Kumar R, Jindal SK	IUATLD based previously standardized questionnaire against physician diagnosis, school based	4723 girls (9 – 20) years	1.9 (frequency 90)
2002	Singh D, Sobti PC, Arora V, Soni RK	Ludhiana population based, house survey using modified ATS criteria for diagnosis	2275 rural children 1 to 15 years	2.6 (frequency 59)
2002	Paramesh H	School survey in 12 schools in the age group of 6 to 15 years	Urban 5570, Rural 990, Total 6560 6 to 15 years children	Group I School in heavy traffic region and children from affluent families: 19.34 Group II School in heavy traffic region and children from less affluent families: 31.14 Group III School in low traffic region and children from affluent families:11.15 Overall Urban: 16.635; Rural: 5.7 Weighted average: 14.97 (frequency 834)
2002	Somasekhar AR, Paramesh H		800, 16 – 19 years adolescents	
2002	Chakravarthy S, Singh RB, Swaminathan S, Venkatesan P	Community-based studies children under 12 years simplified version of the ISAAC questionnaire	855	5.0 (frequency 43)
2004	Singh M, Singh SP, Singh K, Bhatia AS, Kajal NC, Aggarwal D, Singh J	pre-tested, modified, already validated asthma questionnaire	5 – 17 years children Urban: 872 Rural: 758	Urban: 11.92 rural: 13.72 Weighted average: 12.76 (frequency 208)
2004	Awasthi S, Kalra E, Roy S, AwasthiS	Part of multicentric (ISAAC III) trial in Hindi and English, Lucknow, school based	6000 6 – 7 and 13-14 years	2.3 (6 – 7 years) 3.3 (13 – 14 years) Weighted average: 2.8 (frequency 168)
2004	Mistry R, Wickramasingha N, Ogston S, Singh M,	Validated one-page ISAAC questionnaire for Chandigarh	575 13 – 14 years	12.5 (frequency 72)
2005-06	National Family Health Survey (NFHS-3), 2005–06	841 per 100,000 women age 15 – 19 years and 941 per 100,000 men age 15 – 19 years	Boy:841/1lac 15 – 19 years Girl:941/1lac15-19 years girls	0.891 (frequency 89)
2007	Sharma SK, Banga A	Rural school-based studies Hindi translated version of Phase III of the ISAAC questionnaires	8470 6 – 7 years (4128) and 13 – 14 years (4342)	Asthma: 259 (3.0%) 6 – 7 yrs: 150 (3.6%), 13 – 14 yrs: 109 (2.5%) Ever wheezers: 437 (5.16%) 6 – 7 yrs: 232 (5.6%), 3 – 14 yrs: 205 (4.7%) Last 12 mo wheezers: 300 (3.5%) 6 – 7 yrs: 181 (4.4%),13 – 14 yrs: 119 (2.7%) Last 12 mo > 3 per wk wheezers: 92 (1.1%) 6-7 yrs: 66 (1.6%), 13-14 yrs: 26 (0.6%) Awake ≥ 1 night per wk: 67 (0.8%) 6-7 yrs: 46 (1.1%), 13-14 yrs: 21 (0.5%) Wheezing during exercise: 310 (3.6%) 6-7 yrs: 181 (4.2%), 13-14 yrs: 129 (3.1%)

*Ever wheezers = No. of children with history of wheezing ever, Last 12 mo wheezers = No. of children with history of wheezing in the last 12 months, Last 12 mo > 3 per wk wheezers = No. of children with > 3 episodes of wheezing / week in the last 12 months, Awake ≥ 1 night per wk = No. of children with awakening due to wheezing one or more nights/week, Asthma = diagnosed with asthma; Wheezing during exercise = children with wheezing during exercise, **Weighted average prevalence in the studies was calculated from the total sample population in the study divided by all the positive cases, independent of age, wherever overall prevalence data could not be reported in any of the above studies; IUATLD: International Union Against Tuberculosis and Lung Diseases; ISAAC: International study of Bronchial Asthma and allergies in childhood; ATS: American Thoracic Society

The median prevalence from these studies conducted during 1998 – 2004 was determined to be 4.75% (with IQR = 2.65 – 12.35%).

The overall weighted average prevalence was calculated from the total children surveyed in these studies. The total sample population was 295491 (mean 21106.5 and SD 52427.19) and all the positive cases in these 14 studies were 8109 (mean 579.21 and SD 768.48). The mean of the weighted prevalence was found to be 2.74.

Consistent patterns of childhood asthma in Indian children were not found, even after adjusting for these confounding characteristics. Childhood asthma among children 13 – 14 years of age was lower than that in younger children (6 – 7 years of age). Urban and male predominance with wide inter-regional variation in prevalence was observed.

## Discussion

Wide differences in samples, primary outcome variables, lack of consistency in age category, rural-urban variation, criteria for positive diagnosis, and study instruments confounded the outcome variables in our study. The mean prevalence was 7.24 ± SD 5.42. The median prevalence was 4.75% (with IQR = 2.65 – 12.35%). The overall weighted mean prevalence was found to be 2.74. Childhood asthma among children 13 – 14 years of age was lower than that in younger children (6 – 7 years of age). In the socio-demographic analysis urban children had higher general prevalence with male predominance. We observed wide inter-regional variation in prevalence.

The diagnosis of asthma is dependant on the clinical presentation of bronchospasm, variable airway narrowing, bronchial hyper-responsiveness, airway inflammation, and response to inhaled bronchodilators or corticosteroids. Moreover, spirometry results are often normal, and reversibility to bronchodilators is not consistently present. Although numerous epidemiological studies have been carried out all over the world; the magnitude of the problem of asthma has not been defined with certainty. Indeed, bronchial asthma prevalence studies lack consistency, possibly because of the ill-defined diagnostic criteria, non-standardized study protocols, and different methodologies. These have made international and even national comparisons difficult, which incidentally have significant ethnic and regional variations. An increasing morbidity and mortality, as well as healthcare burden from asthma has been recognized lately. In recent years, a majority of the researchers are either using a questionnaire suggested by the 50-nation International Study of Bronchial Asthma and Allergy in Children (ISSAC) or the definition of Bronchial Asthma as modified by the United Kingdom Medical Research Council (MRC). The estimated global prevalence of asthma is 200 million with a mortality of around 0.2 million per year. Although the prevalence is more in developed countries, the developing countries have a higher total burden of the disease due to differences in population. In India, the estimated burden of asthma is believed to be more than 15 million. There was a constant and variable increase in asthma prevalence worldwide in the last two decades and the same is being observed in India.(1,8,21–32)

The ISSAC study compared the prevalence rates of Bronchial Asthma and atopic diseases in 155 centers in 56 countries worldwide, and it was conducted over a period of one year in 7, 21,601 children aged between six and seven years and 13 – 14 years, respectively. Overall, the prevalence of Bronchial Asthma tended to be greater in English speaking countries, but the international pattern suggested that environmental factors may have played a role in the prevalence of Childhood Bronchial Asthma. Evidence from the ISAAC study also showed that the distribution of Childhood Bronchial Asthma varied between global populations from less than 2% to approximately 33%. Prevalence reached 17 – 30% in the UK, New Zealand, and Australia, whereas, areas of low prevalence (1 – 7%) include Eastern Europe, China, and Indonesia. ISAAC phase I reported that a 12-month prevalence of symptoms of wheeze varied between 4.1 and 32.1%, with the lowest rates in India, Indonesia, Iran, and Malaysia, and the highest rates in Australia, Brazil, Costa Rica, New Zealand, and Panama in the age group of six to seven years. For the 13 – 14 year age group, 12-month prevalence of symptoms of wheeze ranged from 2.1 – 4.4% in Albania, China, Greece, Georgia, Indonesia, Romania, and Russia to 29.1 – 32.2% in Australia, New Zealand, Ireland, and UK. The steering committee of ISAAC, in the study on worldwide prevalence of symptoms of asthma, allergic rhino-conjunctivitis, and atopic asthma, in 1998, found 6.0% (Wheeze) and 4.5% (Ever asthma) in India, on the highest ever sample size of 37171, which is similar to our analysis. The prevalence of asthma in Asian countries varies between 5.2% in Taipei to 30% in New Zealand, and in other countries it is around 10 – 17%. The phase I survey was repeated after an interval of 5 – 10 years in 106 centers in 56 countries in children aged 13 – 14 years (n = 304,679) and in 66 centers in 37 countries in children aged six to seven years (n = 193,404). In phase III of the ISAAC study, worldwide trends in the prevalence of asthma symptoms were done recently. Mean symptom prevalence of severe asthma or the symptom prevalence was measured with the asthma video questionnaire. The time trends in asthma symptom prevalence showed different regional patterns. In the Indian subcontinent in children aged 13 – 14 and 6 – 7 years, the prevalence increased per year by +0.02 and +0.06%, respectively Although there was little change in the overall prevalence of the current wheeze, the percentage of children reported to have had asthma increased significantly, possibly reflecting a greater awareness of this condition and/or changes in diagnostic practice. The increases in asthma symptom prevalence in Africa, Latin America, and parts of Asia indicate that the global burden of asthma is continuing to rise, but the global prevalence differences are lessening.(4,21,25)

The outcome of our systematic study was consistent with the prevalence rates of asthma (0.2 – 6.3%) reported from other developing countries.(33–39) Nevertheless, these were quiet high compared to the prevalence of 1966, which was probably the earliest in this series. In that study Vishwanathan R *et al*. reported a prevalence of 0.2% in children below nine years.(7) The same is also valid for comparison with the recent data from the National Family Health Survey (NFHS-3), 2005 – 2006. Although asthma was much more common in women and men than TB, diabetes, or goiter / other thyroid disorders, the reported prevalence was quite low. The number of women with asthma has climbed steadily with age; from 841 per 100,000 women age 15 – 19 to 2,787 per 100,000 women age 35 – 49. Men exhibit a similar progression of asthma by age; men at age 35 – 49 years were three times more likely to have asthma than men at age 15 – 19 years (2,685 versus 941 per 100,000). The number of people with asthma is high in both urban and rural areas, but it is somewhat higher in rural areas (1,719 per 100,000 for women and 1,799 per 100,000 for men).(19)

In our study, we observed that childhood asthma at 13 – 14 years of age was lower than in younger children (6 – 7 years of age). Researchers in the field opined that higher prevalence of asthma in the younger age group was consistent with the widely believed concept of “children growing out of allergic diseases.”(39)

Urban and male predominance with wide inter-regional variation in prevalence was also observed by us in different Indian studies, with a wide variation (4 – 20%) and an increase in mortality in younger age groups. Environmental factors, including increasing exposure to pollution, allergies, tobacco smoke, and sedentary lifestyle were identified as risk factors for asthma. The proportion of Indian school children suffering from Bronchial Asthma had increased to more than double in the last 10 years and reached the highest-level ever. There was low prevalence of Bronchial Asthma (1 – 3.3%) in the children surveyed in Lucknow, Ludhiana, and Punjab, while in Delhi the prevalence of Bronchial Asthma was 11.6%. Rise in prevalence over time in Bangalore had been associated with environmental pollution, urbanization, and change in the demography of the city. These factors might be responsible for inter-city variation in the prevalence of Childhood Bronchial Asthma. Among the children who reported as ever wheezers, one-fourth of them confirmed to be suffering from Bronchial Asthma in later life. The rest of the children with reported wheeze might have either misclassified wheeze or had episodes of lung infection with bronchospasm or an attack of Bronchial Asthma without recurrence. The Bangalore study showed increasing trend of 9 and 29.5% prevalence of asthma in 1979 and 1999, respectively. Boys had a significantly higher prevalence of current asthma as compared to girls (12.8 and 10.7%, respectively). Multiple logistic regression analysis showed that male sex, a positive family history of atopic disorders, and the presence of smokers in the family were significant factors influencing the development of asthma. In a recent study carried out in Delhi 11.9% of the school children in the age group of 5 – 16 years had current bronchial asthma. Significant risk factors for its development were male sex, a positive family history of atopic disorders, and the presence of smokers in the family.(20,25,40–43)

Among those who reported as ever wheezers, almost one-fourth reported asthma. The rest of the children with reported wheeze may have either misclassified wheeze or had episodes of lung infection with bronchospasm or an attack of asthma without recurrence. The study done in urban and rural children in Tamil Nadu in the age group of (6 – 12) years showed prevalence of wheeze 18%.(15)

In an evaluation of the quality of life in Indian children with bronchial asthma, using a disease-specific, locally appropriate questionnaire, the disease-specific QOL score correlated inversely with the symptom score in children with bronchial asthma.(44) The socioeconomic burden of childhood asthma has not been assessed in India. A group of researchers interviewed parents of 85 asthmatic children in the age group of 6 – 17 years, who were suffering from asthma. They revealed the severe burden experienced by the family in 25.9% of the cases, which was significantly associated with the severity of the asthma (*P* < 0.001) and socioeconomic status of the family (*P* < 0.01).(45)

Wheezing is also a significant cause of morbidity among rural children in our neighbouring country Bangladesh.(46) The prevalence rate for wheezing in Galle (28.7%) was higher than in Chandigarh (12.5%), where there were 13 – 14-year-old children living in an old-fashioned, congested city, rather than in a clean and modern city (Galle versus Chandigarh) in South Asia,(18) which indicated scientific urban planning plays a role in prevention of childhood bronchial asthma.

In a recent landmark Indian study, the researchers found a consistent association between being exposed to, and having experienced domestic violence, and childhood asthma prevalence in India. In an age-stratified analysis, a strong association was observed in age groups of under-five, 5 – 14, 15 – 24, and 25 – 44 years. Stress-induced mechanisms, partially captured through violence and social circumstances, may be a missing link in furthering our understanding of the social disparities in asthma.(47) Other studies have also reported higher incidence of psychosocial adaptation problems in children with asthma, particularly severe asthma, than children in the general population. This has been ascribed to adverse developmental impact of having a chronic health problem, increased demands on the family, and dysfunctional familial interactional patterns.(48)

In a study in Canada, asthma morbidity using hospitalization data from 1970 – 1989 in children of Registered Indians in Saskatchewan, showed a significantly increased risk for hospitalization for asthma in children of age 0 – 4 years. Even though asthma was reported to be rare before 1975, it has increased in recent years among Indian children,(49) and points toward genetic potential.

## Conclusion

The World Health Organization recognizes asthma as a major health problem.(50) Still, there is paucity of data on the prevalence of Bronchial Asthma in children in India. Our summary from research studies of Childhood Bronchial Asthma is that Bronchial Asthma affects a large number of children in India and the findings indicate that prevalence of Bronchial Asthma in children in India is increasing at a faster rate than previously understood. Due to lack of national representative data on the prevalence, risk factors, and prognosis of the disease, there is an urgent need for more public health research in this field of priority attention and direction.

### Recommendations

There is a widespread concern that the prevalence and incidence of asthma is still rising in developed countries, but the economic and humanitarian effects of asthma are greater in the developing world, where the prevalence is also rising. The worst sufferer from indoor environment-induced asthma is children in low-income urban families. In addition to direct medical costs, the symptoms experienced by asthmatics lead to absenteeism from school. The burden of asthma affects the patients, their families, and society in terms of lost work and school, lessened quality of life, and avoidable Emergency Department visits, hospitalizations, and deaths.(51–55) Incidentally, the recent KAP study on the newer trends in childhood asthma management in a central Mumbai suburb revealed lesser awareness among family physicians, with no difference based on the number of years in practice. The present modes of information dissemination are insufficient and require supplementation and reinforcement.(56) The International Conference on Health Care Delivery for Asthma, addressing the present status of asthma throughout the world, on how different countries were dealing with the worldwide epidemic of asthma, found that 10% of the citizens have access to the level of care proposed by the guidelines. Asthma has a lower place on the radar screen in a country where 1000 people die each day from tuberculosis. The obstacles to asthma care in India are the costs of care and medications, the socioeconomic disparity within the country, use of multiple languages, cultural issues, and the common use of alternative remedies.(57)
